# Silent infection of human dendritic cells by African and Asian strains of Zika virus

**DOI:** 10.1038/s41598-018-23734-3

**Published:** 2018-04-03

**Authors:** Nathalie J. Vielle, Beatrice Zumkehr, Obdulio García-Nicolás, Fabian Blank, Miloš Stojanov, Didier Musso, David Baud, Artur Summerfield, Marco P. Alves

**Affiliations:** 1 Federal Department of Home Affairs, Institute of Virology and Immunology, Mittelhäusern, Switzerland; 2 Graduate School for Cellular and Biomedical Sciences, University of Bern, Bern, Switzerland; 3 Respiratory Medicine, Department of Clinical Research, Bern University Hospital, University of Bern, Bern, Switzerland; 4 Materno-fetal and Obstetrics Research Unit, Department Woman-Mother-Child, Lausanne University Hospital, Lausanne, Switzerland; 5 Unit of Emerging Infectious Diseases, Institut Louis Malardé, Papeete, Tahiti French Polynesia; 6 Department of Infectious Diseases and Pathobiology, Vetsuisse Faculty, University of Bern, Bern, Switzerland; 7 Aix Marseille University, IRD, AP-HM, SSA, VITROME, IHU-Méditerranée infection, Marseille, France

## Abstract

While Zika virus (ZIKV) circulated for decades (African lineage strains) without report of outbreaks and severe complications, its emergence in French Polynesia and subsequently in the Americas (Asian lineage strains) was associated with description of severe neurological defects in newborns/neonates and adults. With the aim to identify virus lineage-dependent factors, we compared cell susceptibility, virus replication, cell death and innate immune responses following infection with two African and three contemporary Asian lineage strains of ZIKV. To this end, we used green monkey Vero and *Aedes albopictus* C6/36 cells and human monocyte-derived dendritic cells (DCs). The latter are involved in the pathogenesis of several mosquito-borne *Flavivirus* infections. In Vero and C6/36 cells, we observed strain- but not lineage-dependent differences in infection profiles. Nevertheless, in human DCs, no significant differences in susceptibility and virus replication were found between lineages and strains. ZIKV induced antiviral interferon type I/III in a limited fashion, with the exception of one African strain. None of the strains induced cell death or DC maturation in terms of MHC II, CD40, CD80/86 or CCR7 expression. Taken together, our data suggest that a large collection of virus isolates needs to be investigated before conclusions on lineage differences can be made.

## Introduction

Zika virus (ZIKV) is an emerging mosquito-borne *Flavivirus* of the *Flaviviridae* family causing congenital ZIKV syndrome, including microcephaly, and severe neurological complications in adults^1,2^. Originally, the virus was isolated from a rhesus macaque in 1947 in the Zika Forest of Uganda^3^. ZIKV is mainly transmitted by mosquitoes of the *Aedes* genus such as *A. aegypti*. The first human case of ZIKV infection was probably reported in 1962/63 in Uganda^4^. Since then, the virus has spread to Southeast Asia and the Pacific Islands of Yap in the Federated States of Micronesia, where the first large ZIKV outbreak in humans was declared in 2007^5,6^. A second large outbreak occurred in French Polynesia in 2013/2014 and subsequently the virus spread in other Pacific Islands and in the Americas in 2015. In 2012, two lineages of ZIKV were identified, namely the African and the Asian lineages^7^. The virus strains circulating in the Americas, Caribbean, Southeast Asia and Pacific belong to the Asian lineage^8^. Before its emergence in French Polynesia, ZIKV infections were described as asymptomatic or mild dengue-like illness without complications. The new patterns of ZIKV infection include severe neurological complications in adults, mainly Guillain-Barré syndrome (GBS), and in newborns/neonates, Zika congenital syndrome^9,10^. Non-vector borne transmission of ZIKV also has been reported including materno-fetal, sexual and transfusion transmission^8^.

The association of ZIKV infection and microcephaly has been reported in the Americas and retrospectively in French Polynesia^10^. It was not reported before the emergence of ZIKV on large scale, possibly because previous outbreaks were too small and the virus never spread to such a large immunologically naïve population. It is also possible that ZIKV became more pathogenic due to an adaptation to the human host^11^. Despite the apparent difference in pathogenicity between Asian and African lineage ZIKV, most of the studies have been focused on Asian ZIKV strains or on the original African MR766 strain and data showing strain comparison is limited^12^.

Innate immune response to viral infection depends primarily on the functions of dendritic cells (DCs) and macrophages. DCs form a heterogeneous group of antigen presenting cells described as sentinels of the immune system and recognize viruses at their sites of entry, for example, in the skin at the site of a mosquito bite but also at mucosal surfaces^13,14^. Monocytes, which circulate in the blood, can differentiate into monocyte-derived DCs (MoDCs) during inflammation^15^. This subset of DC is also found at the body surfaces in contact with the environment^16^. Recently, it has been shown that ZIKV is capable of infecting monocytes and placenta-specific macrophages, namely Hofbauer cells^17,18^. Macrophages are closely related to DCs and both cell types have central roles in the pathogenesis of flaviviruses such as Dengue virus (DENV) and West Nile virus (WNV)^19,20^. Since many pathogenic viruses specifically interfere with DC functions, this cell type is central towards viral infection outcomes. They express a number of pattern recognition receptors (PRRs) such as Toll-like receptors (TLRs) that lead to the activation of the interferon (IFN) pathway and subsequent transcription of interferon-stimulated genes (ISGs) such as Viperin (virus inhibitory protein, endoplasmatic reticulum associated, IFN-inducible), MxA (Myxovirus resistance gene A), and 2′,5′-OAS (2′,5′-oligoadenylate synthase)^21,22^. The role of type I IFNs is essential in defense against viral pathogens. Additionally, type III IFN (IFN-λ1/2/3) may play a role in antiviral response, particularly at body surfaces such as the skin and the genital tract^23,24^. In addition, IFN-λs are produced by DCs upon infection with DENV and other RNA viruses and seem to play a role in neuroinvasion of WNV^25–27^. In regard to ZIKV infection, mechanisms of IFN signaling antagonism have been proposed via the non-structural protein 5 (NS5), highly conserved among flaviviruses, in human embryonic kidney cells and placenta-specific cells^28^. Also, evasion mechanisms of WNV and DENV include repression of type I IFN pathway mediated by viral noncoding subgenomic flavivirus RNA (sfRNA)^29,30^. sfRNA is a product of enzymatic RNA degradation of 3′ untranslated region of the viral genome by the cellular exoribonuclease XRN1. It has been shown that sfRNA of DENV and WNV plays a role in inhibiting IFN responses, opening new questions on similarities with newly emerging ZIKV^30,31^.

Severe neurological complications such as GBS and microcephaly have never been described during infections of humans with the African lineage ZIKV. Consequently, it is tempting to think that spreading of the virus to immune privileged sites such as the brain and the placenta and the triggering of an effective immune response could depend on which lineage the ZIKV strains belongs to. First of all, we aimed at comparing replication parameters of several ZIKV strains of the African and the Asian lineage in a simple cellular system. Thus, we evaluated susceptibility, live virus release, viral loads, and cytopathic effect of two African and three contemporary Asian ZIKV strains in two cell lines routinely used for the virus propagation and titration, namely African green monkey kidney Vero and *A. albopictus* C6/36 cells. Next, we tried to extrapolate our findings by identifying potential differences between African and Asian lineages in a relevant cell type involved in flavivirus pathogenesis. Indeed, as a more physiological cellular model of ZIKV infection, we analyzed infection parameters and IFN pathway induction at the level of IFNs and ISGs in human MoDCs. The levels of sfRNA produced upon infection of MoDCs by the different ZIKV strains tested have also been evaluated.

## Results

### Strain-specific infection profiles in Vero and C6/36 cells

**Figure 1:**
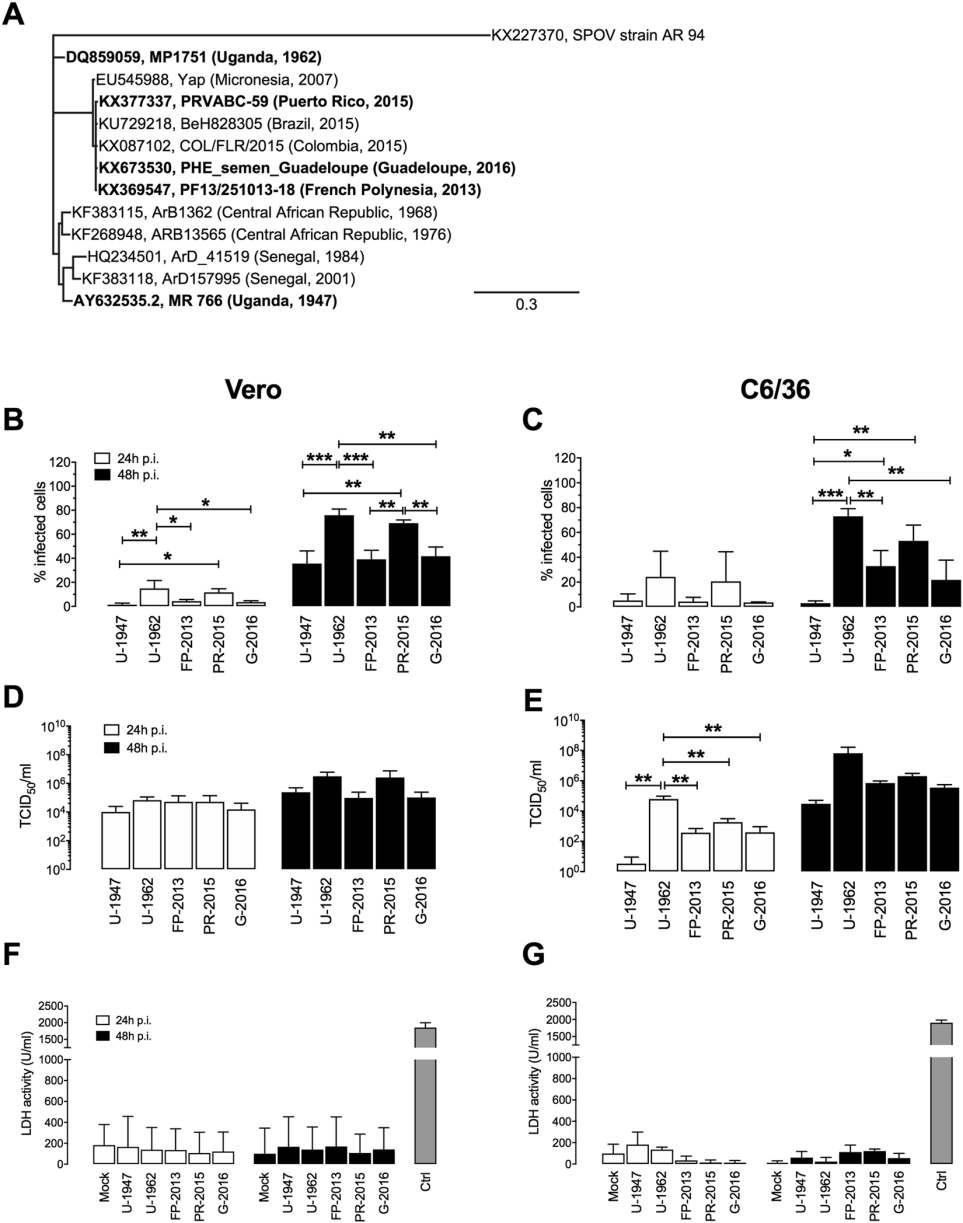
Susceptibility of Vero and C6/36 cells towards African and Asian ZIKV strains infection. Phylogenetic analysis of the African and Asian ZIKV strains used in this study (highlighted in bold) (**A**). For each sequence, accession number, strain, and country and year of isolation are shown. Scale bar represents the number of substitutions per nucleotide position. Flow cytometry analysis of the frequency of ZIKV-infected Vero cells (**B**) 24, and 48 h p.i. and C6/36 cells (**C**) 24 h, and 48 h p.i. with a MOI of 0.1 TCID_50_/cell. Measurement by TCID_50_ assay of infectious ZIKV release in supernatants of Vero cultures 24 and 48 h p.i. (**D**) and C6/36 cultures 24, and 48 h p.i. (**E**) with ZIKV at a MOI of 0.1 TCID_50_/cell. LDH activity measurements of virus-induced cell death in supernatants of Vero (**F**) and C6/36 (**G**) cultures. Supernatant of dead cells obtained by incubating at 65 °C for 15 minutes was used as a positive control. The data shown are representative of 3 independent experiments, data are presented as mean +/− SD. Differences among strains were tested with one-way ANOVA with Tukey correction for multiple comparisons. Stars indicate significance levels; *p < 0.05, **p < 0.01, ***p < 0.001.

We used Vero and C6/36 cells as standard cell types for flavivirus work to evaluate the infection profiles of ZIKV strains of the African and the Asian lineages. The prototypic strains MR766 (from here referred as “U-1947”), originally isolated from a sentinel monkey, and MP1751 (referred as “U-1962”), isolated from a pool of mosquitoes (*A. africanus*), were used^3,32^. The emerging ZIKV strains used were isolated from human patient specimens infected in French Polynesia (PF13/25013-18, referred to as “FP-2013”), in Puerto Rico (PRVABC59, referred to as “PR-2015”) and in Guadeloupe (PHE_semen_Guadeloupe referred to as “G-2016”) (Fig. 1A). Of note, although the U-1947 and U-1962 strains belong to the African lineage of ZIKV, they cannot be considered as optimal representatives of this lineage due to long passage history (U-1947) and the relatively distant phylogenetic relation with the African lineage group (U-1962) (Fig. 1A). However, due to the difficulty in obtaining African isolates, these particular two strains have been widely used and are thus relevant to allow comparison with previous studies. For both cell types, there was an increase in the percentage of infected cells over time. The low passage African U-1962 and the Asian PR-2015 strains showed the highest rates of infection 24 and 48 h p.i. More specifically, the frequency of infected Vero cells was significantly higher after infection with U-1962 24 and 48 h p.i. when compared to the strains U-1947 (p < 0.001 at 48 h p.i.), FP-2013 (p < 0.001 at 48 h p.i.) and G-2016 (p < 0.01 at 48 h p.i.). As for the U-1962 strain, we observed significantly higher infection rates of Vero cells 48 h p.i. with PR-2015 in comparison to U-1947 (p < 0.01), FP-2013 (p < 0.01), and G-2016 (p < 0.01) strains (Fig. 1B). Similarly to Vero cells, the frequency of infected C6/36 cells was significantly higher after infection with U-1962 when compared to the strains U-1947 (p < 0.001), FP-2013 (p < 0.01), and G-2016 (p < 0.01) 48 h p.i. (Fig. 1C). While not significant, we observed a trend towards a higher infection rate of C6/36 cells with the Asian lineage PR-2015 in comparison to FP-2013, and G-2016 strains (Fig. 1C). In line with its high passage history in mammalian cells, U-1947 showed significantly lower infection rates in C6/36 cells in comparison to the U-1962 (p < 0.001), FP-2013 (p < 0.05), and PR-2015 (p < 0.01) strains 48 h p.i. (Fig. 1C). In order to compare the levels of live ZIKV release in Vero and C6/36 cells, we determined viral titers in supernatants 24 and 48 h p.i. Infectious virus release by both cell types increased between 24 and 48 h p.i., reaching comparable titers 48 h p.i., although the increase was more pronounced for the C6/36 cells particularly at 24 h p.i. with C6/36 cells infected with U-1962 in comparison to all the other strains tested (p < 0.01). Since there is evidence of ZIKV-induced cell death *in vitro*, particularly in neuroprogenitor cells^33^, we investigated cell death response of Vero and C6/36 upon ZIKV infection. The cytopathic effect induced following infection of Vero and C6/36 cells with the African and Asian ZIKV strains was not significantly different to the mock controls (Fig. 1F, G). Together, our data suggest that the differences observed in Vero and C6/36 cells when comparing infectivity, live virus release and cellular death are specific to each strain.

### Human MoDCs show similar susceptibility to African and Asian ZIKV strains

**Figure 2:**
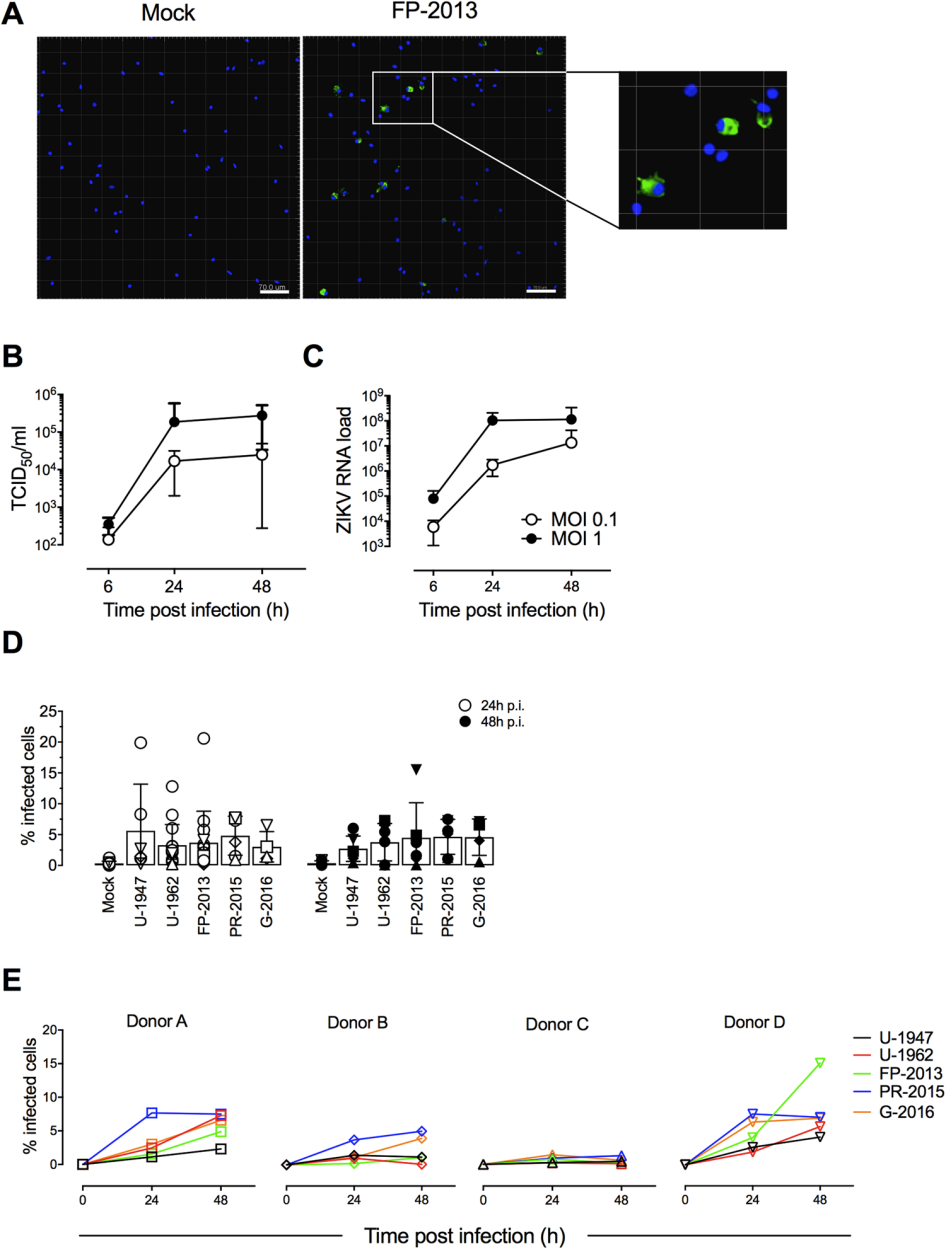
Susceptibility of MoDCs towards African and Asian ZIKV strains infection. (**A**) Confocal microscopy analysis 24 h p.i. with Mock and Asian (FP-2013) lineage ZIKV at a MOI of 1 TCID_50_/cell. Blue: DAPI, Green: ZIKV, bar 70 µm. The micrographs shown are representative of ZIKV-infected DCs generated from 3 independent blood donors. (**B**) Measurement by TCID_50_ assay of infectious ZIKV release in supernatants of MoDC cultures 6, 24, and 48 h p.i. with FP-2013 at a MOI of 0.1 and 1 TCID_50_/cell. (**C**) Measurement of intracellular viral RNA copies by quantitative RT-PCR in MoDC cultures 6, 24, and 48 h p.i. with FP-2013 at a MOI of 0.1 and 1 TCID_50_/cell. Data are presented as mean +/− SD of MoDCs generated from 6 independent blood donors. (**D**) Frequency of ZIKV-infected MoDCs per donor 24, and 48 h p.i. with five ZIKV strains a MOI of 0.1 TCID_50_/cell. Data are presented as mean +/− SD of MoDCs generated from 4–15 independent blood donors. Individual donors are represented with a symbol: square (Donor A), diamond (Donor B), triangle (Donor C), inverted triangle (Donor D). (**E**) Frequency of ZIKV-infected MoDCs for 4 selected donors 24, and 48 h p.i. with five ZIKV strains a MOI of 0.1 TCID_50_/cell.

**Figure 3:**
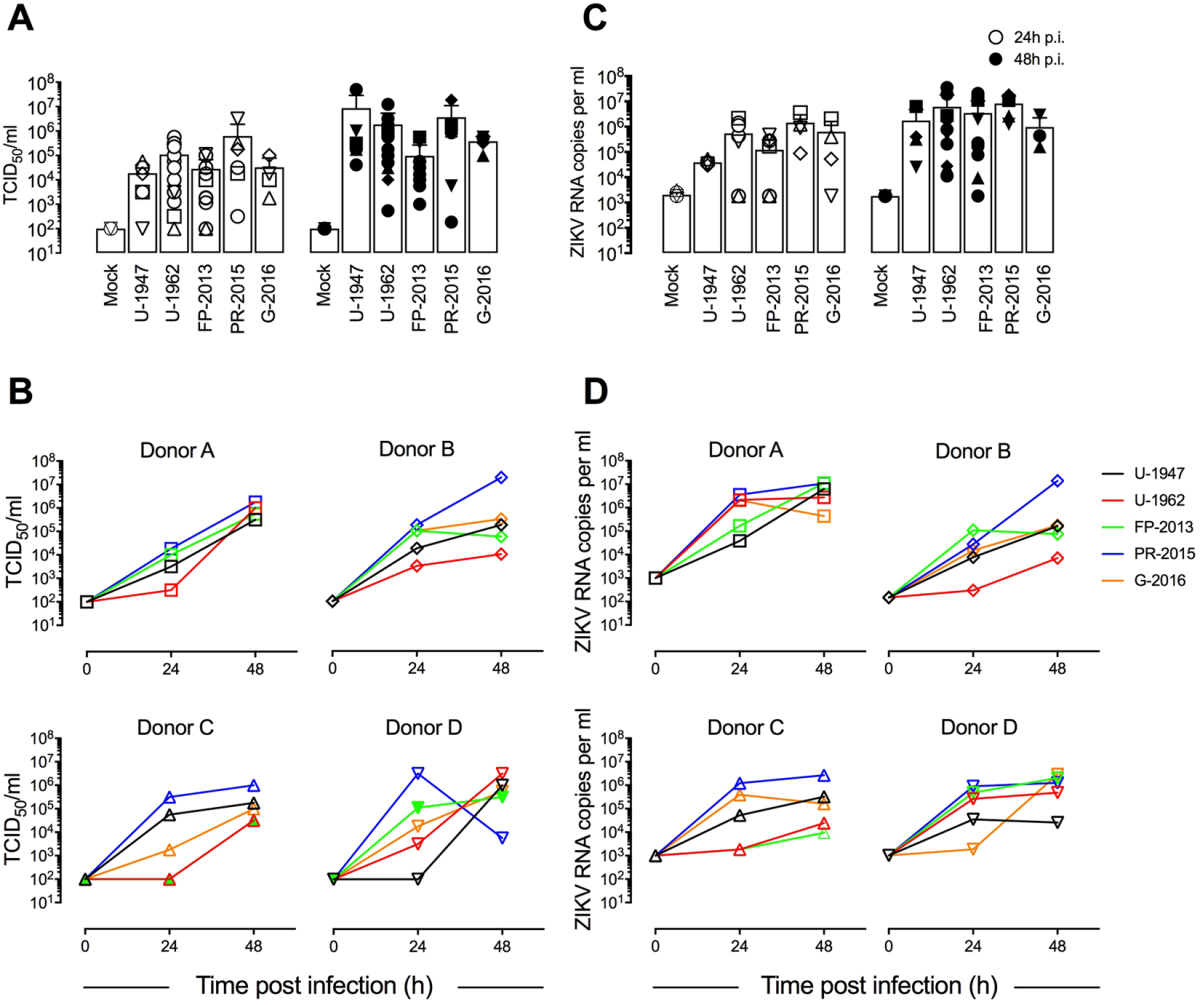
Replication of ZIKV in human MoDCs. (**A**) Measurement by TCID_50_ assay of infectious ZIKV release in supernatants of MoDC cultures 24, and 48 h p.i. with five ZIKV strains at a MOI of 0.1 TCID_50_/cell. Data are presented as mean +/− SD of DCs generated from 4–12 independent blood donors. Individual donors are represented with a symbol: square (Donor A), diamond (Donor B), triangle (Donor C), inverted triangle (Donor D). (**B**) Single donor-dependent variation in infectious ZIKV release in supernatants of MoDC cultures 24, and 48 h p.i. with five ZIKV strains at a MOI of 0.1 TCID_50_/cell. (**C**) Measurement of extracellular viral RNA copies by quantitative RT-PCR in MoDC culture supernatants 24, and 48 h p.i. with five ZIKV strains at a MOI of 0.1 TCID_50_/cell. Data are presented as mean +/− SD of DCs generated from 4–12 independent blood donors. (**D**) Single donor-dependent variation of extracellular viral RNA copies by quantitative RT-PCR in MoDC cultures 24, and 48 h p.i. with five ZIKV strains at a MOI of 0.1 TCID_50_/cell.

Based on our findings on infection of cell lines, we evaluated the susceptibility of human MoDCs to ZIKV strains of the African and the Asian lineages. We used MoDCs generated from independent blood donors to evaluate ZIKV infection rates. The cell surface phenotype of generated MoDCs was found high in CD11c, and negative in CD14, CD3 CD56, and CD19 (Supplementary Fig. S1A). In order to define the experimental conditions to compare the interaction of all the ZIKV strains selected with MoDCs, we conducted preliminary experiments with one ZIKV strain, namely the contemporary strain FP-2013. The ability of ZIKV to infect human MoDCs was first shown by visualization of the ZIKV E protein by confocal imaging. At a MOI of 1.0 TCID_50_/cell 24 h p.i., MoDCs where found to be permissive towards ZIKV infection and a cytosolic localization of the virus was observed (Fig. 2A). Live virus release (Fig. 2B) and viral RNA loads (Fig. 2C) both increased over time, reaching the peak of replication at 24 h p.i. for a MOI of 1 TCID_50_/cell. In order to follow ZIKV over several replication cycles, we decided to focus our investigations on MoDCs challenged with low MOIs (0.1 TCID_50_/cell) and late time points (24 and 48 h p.i.). We further evaluated the frequency of ZIKV-infected MoDCs and didn’t observe significant differences between the ZIKV strains at the times p.i. tested. Indeed, the average percentage of infected MoDCs was comprised between 2.7 and 5.6% among the strains tested and no significant difference was observed between 24 and 48 h p.i. (Fig. 2D). Also, a strong variability in the susceptibility of MoDCs towards ZIKV infection was observed between the donors (Fig. 2E). In order to compare the infectivity of the five ZIKV strains in MoDCs, we evaluated the infectious virus release by determining viral titers in supernatants at 24 and 48 h p.i. In contrast to the low percentage of infected MoDCs over time, the measurements showed a clear increase in the release of infectious viral particles for all strains at both time points after infection, indicating active replication of ZIKV in MoDCs (Fig. 3A). In line with live virus release, we observed an increase over time in extracellular viral RNA loads of the five strains tested (Fig. 3B). Again, we observed a high variability among donors both at the level of live virus release (Fig. 3C) and extracellular viral RNA loads (Fig. 3D). While further investigation is required, our results suggest no lineage-specific differences towards ZIKV infection of MoDCs.

### ZIKV infection does not induce cell death and activation/maturation of MoDCs

**Figure 4:**
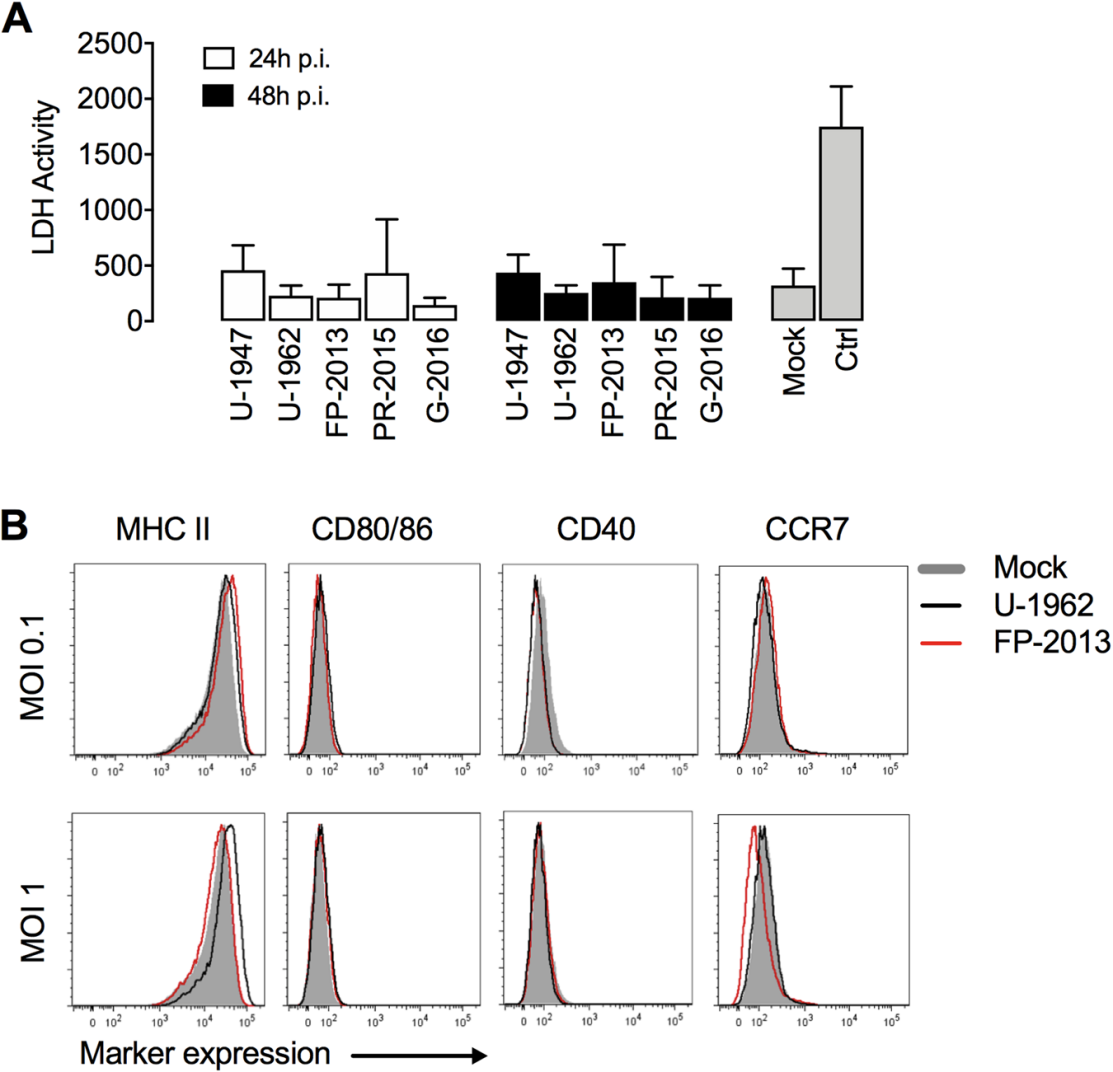
ZIKV infection is not inducing cell death nor activation of MoDCs. (**A**) Cytopathic effect of ZIKV infection of MoDCs assessed by LDH activity 24, and 48 h after mock-treatment or infection with five ZIKV strains at a MOI 0.1 TCID_50_/cell. Supernatant of dead cells was used as a positive control. Data are presented as mean of MoDCs generated from 4 independent blood donors. (**B**) Expression levels of the activation markers MHC class II, CD80/86, CD40 and CCR7 measured by flow cytometry upon MoDCs mock-treated or infected with U-1962 and FP-2013 at a MOI of 0.1 and 1 TCID_50_/cell. The data shown are representative of MoDCs generated from 3 independent blood donors.

In order to determine the cell death response of MoDCs to ZIKV infection, we performed an LDH assay 24 and 48 h p.i. At the time points and MOI tested, we did not observe clear signs of cell death of the cells challenged with ZIKV as well as in mock-infected cells. This result was consistently found in MoDCs generated from 3-4 different blood donors (Fig. 4A). We obtained similar results by using a live/dead assay by flow cytometry (Supplementary Fig. S1B and C). In addition, we measured typical DC activation/maturation markers such as MHC class II, CD80/86, CD40, and CCR7. These markers were found to be expressed at similar levels to mock-treated MoDCs for U-1962 and FP-2013 strains for both MOIs tested (Fig. 4B).

### African and Asian ZIKV strains induce limited expression of type I and III IFNs in MoDCs

**Figure 5:**
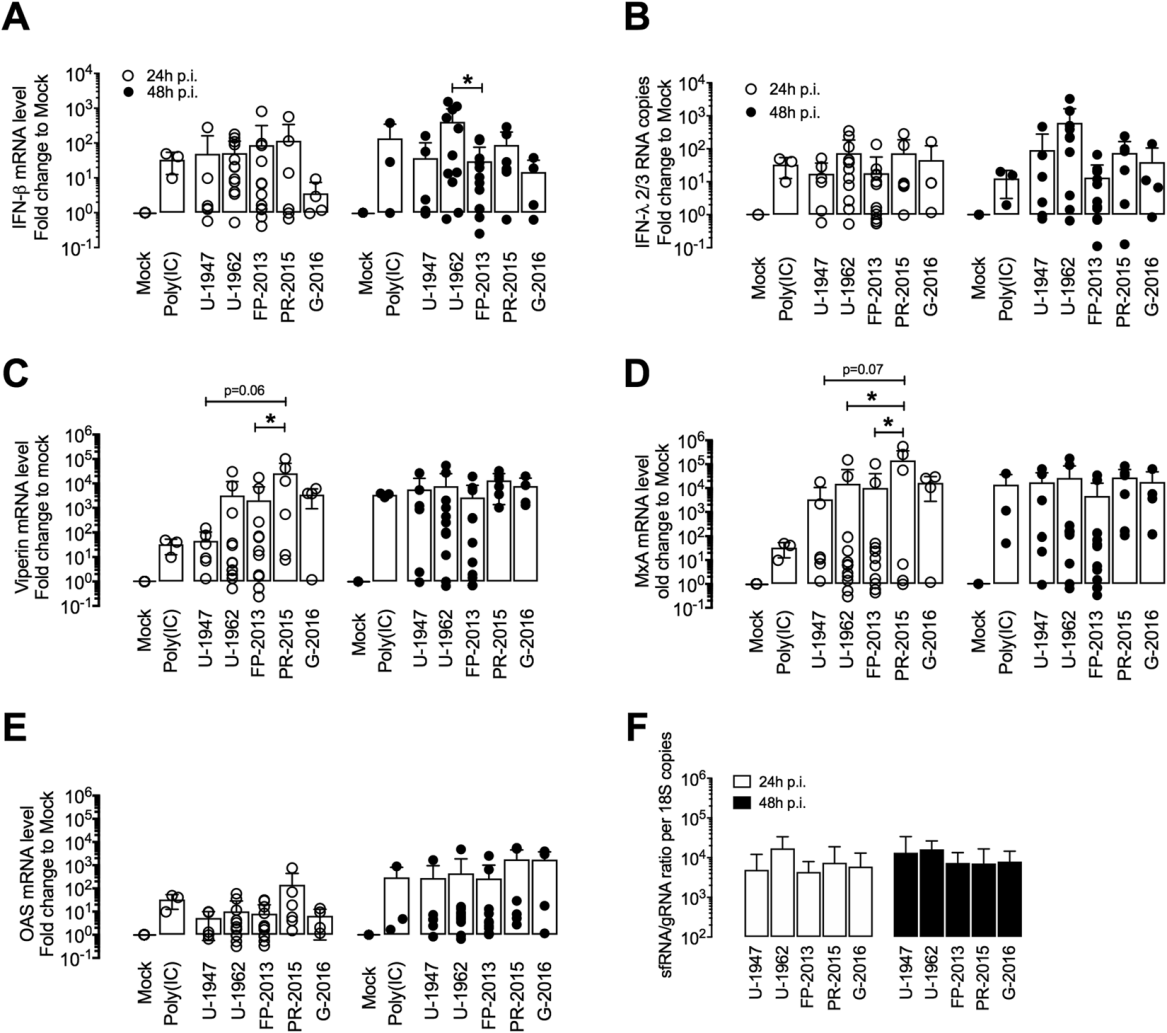
IFN type I/III and ISG responses induced by MoDCs upon ZIKV infection. Fold change of gene expression levels relative to mock of IFN-β (**A**) and IFN-λ2/3 (**B**) measured by quantitative RT-PCR in MoDCs 24, and 48 h p.i. with five ZIKV strains at a MOI of 0.1 TCID_50_/cell and after addition of Poly(IC) and Resiquimod. Fold change of gene expression levels of selected ISGs, namely Viperin (**C**), MxA (**D**), and 2′,5′-OAS (**E**) in MoDCs infected with five ZIKV strains at a MOI of 0.1 TCID_50_/cell and after addition of Poly(IC) and Resiquimod. (**F**) Measurement of sfRNA to gRNA ratios by quantitative RT-PCR in MoDC cultures 24, and 48 h p.i. with five ZIKV strains at a MOI of 0.1 TCID_50_/cell. Data are presented as mean +/− SD of MoDCs generated from 4–12 independent blood donors. Differences among strains were tested with one-way ANOVA with Tukey correction for multiple comparisons. Stars indicate significance levels; *p < 0.05.

Virus-infection of MoDCs is leading to the activation of antiviral innate immune mechanisms, with the IFN type I (IFN-α/β) pathway being of central importance to protect the host. We compared the IFN responses of MoDCs infected with ZIKV strains of the African and the Asian lineages at the mRNA and protein levels. We choose Poly(IC) as a positive control for inducing IFN response and downstream ISG expression over specific RIG-I (retinoic acid-inducible gene I) and TLR-3 agonists, since it induced higher levels in our MoDC cultures (Supplementary Fig. S2). U-1962 induced a significantly higher IFN-β mRNA expression compared to mock than the other strains at 48 h p.i. (Fig. 5A). This higher level of expression was statistically significant when comparing with FP-2013 48 h p.i. (p < 0.05). Importantly, IFN-α mRNAs and the protein levels of IFN-α/β were found to be undetectable by RT-PCR and ELISA respectively upon infection with the U-1962 and FP-2013 strains (n = 6). The IFN-λ2/3 mRNA expression did not mirror the IFN-β expression patterns at 24 h p.i. but at 48 h p.i a similar trend was observed (Fig. 5B). Furthermore, we assessed the level of expression of three ISGs. In line with the IFN responses, MoDCs infected expressed highly donor-dependent levels of Viperin (Fig. 5C), MxA (Fig. 5D) and 2′,5′-OAS (Fig. 5E). We measured statistically significant differences in Viperin expression between FP-2013 and PR-2015 (p < 0.05), and a trend towards significance between U-1947 and PR-2015 (p = 0.06). For MxA induction, there was a significant difference between PR-2015 and the two strains FP-2013 and U-1962 (both p < 0.05), and a trend towards significance with U-1947 (p = 0.07). The expression of RIG-I-like receptors (RLRs) upon infection with ZIKV was also upregulated but we could only observe a statistical difference between the U-1947 and PR-2015 strains for MDA-5 (Supplementary Fig. S3). Finally, in order to compare the induction of sfRNA by the different ZIKV strains, we measured the ratios of ZIKV sfRNA and genomic RNA (gRNA) expressed in MoDCs, 24 and 48 h p.i. At both time points, there were no significant differences between the ZIKV strains tested. Specifically, we didn’t observe significantly higher levels of sfRNA and gRNA ratios for the strain leading to a higher induction of an IFN response (U-1962), suggesting the presence of other mechanisms involved in IFN pathway inhibition (Fig. 5F). Alignment of full-length NS5 proteins of U-1962 showed polymorphism compared to the other strains used in this study but none of the varying residues are putative STAT2 binding residues, suggesting that levels of IFN expression are independent of mutations in the NS5 sequence of the U-1962 strain^28^ (Supplementary Fig. S4).

## Discussion

In this study, we made several observations by comparing distinct ZIKV strains of the African and the Asian lineages in different *in vitro* settings. First, we observed strain-related susceptibility of the vertebrate Vero cell line towards African and Asian ZIKV strains and compared it to the invertebrate C6/36 cell line, both commonly used for ZIKV propagation. Second, we analyzed the infection profiles of human MoDCs infected with ZIKV and measured IFNs response at the level of IFN-β, IFN-λs and ISGs and didn’t noticed lineage-dependent differences. Finally, to investigate mechanisms of ZIKV-induced IFN pathway inhibition, we measured the levels of ZIKV sfRNA and observed comparable levels between strains.

In comparison to MoDCs, we measured higher infection rates in Vero and C6/36 cells challenged with several ZIKV strains but similar live virus release. Indeed, unlike with infected Vero and C6/36 cells, the percentage of infected MoDCs did not follow over time ZIKV replication assayed by live virus titration and RT-PCR. This observation suggests that the translation of cell line-based findings of ZIKV biology to more complex physiological systems should be done with precaution. Susceptibility of human MoDCs towards U-1947 strain and currently circulating PR-2015 strain has been previously observed by Bowen *et al*.^34^. Interestingly, in their setting, the PR-2015 strains showed lower rates of infection compared to U-1947. In our study, regardless of the cell type investigated, the PR-2015 isolate showed higher rates of infection and live virus release compared to the other strains of the Asian lineage. This discrepancy may be due to differences in cell infection protocol or virus stock preparation. Similarly to Bowen *et al*., we observed high variations between MoDCs generated from different blood donors^34^. In line with this, when the susceptibility of placenta-specific macrophages was compared with placental fibroblasts, differences in the frequency of infected cells was also observed between cell types and donors^35^. In order to evaluate the donor variability to ZIKV infection *in vitro*, it would be interesting to repeatedly test the susceptibility of MoDCs obtained from the same blood donors. Indeed, successive measurements could potentially lead to the identification of host factors modulating ZIKV infection.

Induction of cellular death is an important mechanism of antiviral response preventing viral replication and dissemination. We did not observe significant cell death after infection of Vero and C6/36 cells, and MoDCs with five ZIKV isolates 48 h p.i. This is in line with previous data on cytopathogenicity of ZIKV *in vitro* showing no significant cellular death of infected human MoDCs with four different ZIKV strains at 48 h p.i. with higher MOIs than used in our study^34^. The available studies investigating the cytopathic effect of African and Asian ZIKV strains *in vitro* are puzzling and seem to be dependent on the cellular target evaluated. Indeed, studies investigating virulence of ZIKV in human neural cells show a lower cytopathic effect of Asian compared to African lineage strains^36,37^. In addition, human endometrial stromal cells showed higher cell-death response after infection with U-1947 compared to a contemporary Asian strain^38^. However, in an infection model of vascular endothelial cells with a circulating PR-2015 isolate and U-1947 strain, the authors observed that the circulating Puerto Rican isolate is inducing stronger cell death and faster viral RNA replication rates compared to an African isolate^39^. In a recent study, Dowall *et al*. challenged mice carrying a deficiency in type-I IFN receptor with the strains U-1962, PR-2015 and G-2016. The selected strain from the African lineage induced histological changes, weight loss and death, whereas the Asian strains induced neither weight loss nor death and only one animal showed mild histological changes. The investigation has been carried out by using only one African strain making the primary conclusion of the study, that there are lineage-specific differences, delicate to fully validate^40^. Consequently, we question lineage-specific induction of cellular death and rather speculate that previous reports on various cell types were possibly describing strain-specific differences.

When DCs become activated after having recognized a pathogen, they produce inflammatory and antiviral cytokines and migrate to the draining lymph nodes^41^. DC maturation is characterized by the increase in expression of MHC class II and co-stimulatory markers such as CD80/86 which are required for efficient priming of T-cell response^42^. We did not observe increased expression of neither MHC class II nor CD80/86 in our MoDC cultures upon infection with both ZIKV lineages, as previously shown by others^34^. There seems to be important differences when looking at various flaviviruses and their strategies to circumvent host immunity. Diminution of DC activation/maturation has been proposed for ZIKV, tick-borne encephalitis virus as well as DENV, where virus-exposure of DCs blocks activation and maturation of infected cells. DENV-infected MoDCs in contrast to non-infected bystander DCs do not upregulate MHC molecules as well as co-stimulatory molecules CD80/86^34,43,44^. Taken together, the low cytopathic effect of a ZIKV strain and its ability to interfere with MoDC activation and maturation could result in delayed immune responses and help in the understanding of its capacity to reach immune-privileged sites.

To evaluate antiviral mechanism in play during ZIKV infection of MoDCs, we compared type I and III IFN responses towards five ZIKV strains. Since ZIKV pathogenesis involves placenta barrier-crossing, Bayer *et al*. highlighted the importance of type III IFNs release by primary human trophoblasts for defense against ZIKV^45^. Moreover, Bowen *et al*. showed that human MoDCs infected with ZIKV released minimal levels of type I and type III IFNs^34^. In our setting, the IFN responses induced by the various strains were comparably low although the low-passage U-1962 induced significantly higher levels of IFN-β and a trend towards higher levels of IFN-λ2/3. We measured expression levels of selected ISGs commonly induced upon viral infections, which confirmed our previous findings regarding type I IFN expression. Indeed, levels of Viperin, MxA and 2′,5′-OAS expressed after infection with ZIKV were similarly expressed by all strains. When looking at type III IFN expression, the African isolate U-1962 showed the strongest induction. Interestingly, U-1962 is a low-passage insect-isolated strain and seems to be a better inducer of IFN compared to mammalian-isolated strains. While displaying similar replication profiles in MoDCs in comparison with other strains, the U-1962 strain is inducing a stronger antiviral response at the level of IFNs. Thus, this particular strain could represent a valuable tool to assess the antagonizing mechanisms used by ZIKV to bypass the innate immune response of the host. Dealing with immune evasion mechanisms used by ZIKV, Donald *et al*. described the presence of sfRNA in Asian lineage ZIKV-infected Vero cells and its type I IFN antagonism activity^46^. Furthermore, recent data has shown the capacity of a circulating Brazilian ZIKV strain to inhibit type I IFN induction at the level of RIG-I via the production of sfRNA^46,47^. In our setting, we found no differences in sfRNA expression in MoDCs which could explain differential patterns of IFN and ISG expression. In regards to NS5 and its role in IFN signaling pathway inhibition, we aligned the protein sequences of the strains used in this study^28^. We found seven amino acids in the U-1962 NS5 sequence which differ from the other strains. Nevertheless, none of them are putative STAT2 binding residues, which goes against the idea of the link between NS5 polymorphism and IFN pathway inhibition^48^. Studies on epidemiological fitness of flaviviruses such as DENV and WNV showed that the ability of a viral strain to inhibit IFN response plays a role in the epidemiological dominance of a strain^29,30,49^. Thus, the potent antagonizing mechanisms of emerging ZIKV strains leading to interference with the IFN pathway require careful consideration and investigation.

In summary, we observed in Vero and C6/36 cells significant strain differences in the frequency of ZIKV infection and to a lower extent in live virus release. However, when evaluating the interaction of ZIKV strains of both lineages with primary MoDCs, a relevant model mimicking first line immune response, no significant differences between the lineages and strains were found. Our data show that while weakly susceptible towards ZIKV strains infection, DCs are exploited by the virus to release high levels of infectious particles and viral RNA. Remarkably, despite triggering an IFN response, ZIKV didn’t induce cell death response nor activation/maturation of DCs, pointing on immunological evasion mechanisms. Taken together, our data suggest that the apparent epidemiological differences between the African and Asian ZIKV lineages are not recapitulated in the relevant primary MoDC culture system and that a large collection of virus isolates needs to be investigated before conclusions on lineage differences can be made. Undoubtedly, more work is required to understand the contribution of viral and environmental factors in the recent emergence of severe neurological outcomes during ZIKV infection.

## Material and Methods

### Ethics Statement

Buffy coats from healthy individuals were obtained from anonymous blood donors by the Swiss Transfusion SRC (Swiss Red Cross) Inc. (Regional transfusion blood service, Bern, Switzerland). The use of buffy coats was approved by the Swiss Transfusion SRC Institutional review board. All experimental protocols were approved and performed according to the institution’s guidelines.

### Virus strains propagation

The high passage original African lineage MR766 strain (U-1947; GenBank AY632535.2) was obtained from American Type Culture Collection (ATCC). The passage history includes numerous passages in suckling mice (>100) followed at least by one passage in Vero cells^3^. The low passage African lineage strain MP1751 (U-1962; Genbank DQ859059) has been isolated from a pool of *A. africanus* in 1962 and has been passaged up to four times between 1962 and 1972 by an unknown method followed by one passage in Vero cells in 2011 (PHE). The low passage ZIKV clinical isolates (<5 passages) of the Asian lineage were obtained from viremic patients in French Polynesia in 2013 (FP-2013; PF13/25013-18; GenBank KX369547), and in Puerto Rico in 2015 (PR-2015; PRVABC59; GenBank KX377337) and in Guadeloupe (G-2016; PHE_Semen_Guadeloupe; GenBank KX673530) in 2016 (the two later obtained from PHE). All the five strains were passaged identically twice to generate working stocks and back titrated in our facility in *A. albopictus* C6/36 cells, cultured in G-MEM-BHK-21 (Gibco) supplemented with 2% FBS (Biochrom). A MOI of 0.02 TCID_50_/cell was used and C6/36 cell supernatants were harvested after 96 h of incubation at 28 °C. The virus stocks were titrated on Vero cells (CCL-81, ATCC) cultured in MEM (Gibco) supplemented with 2% FBS (Biochrom). Of note, the ZIKV strains providers have certified the identity of the PR-2015, G-2016, and U-1947 strains (PHE and ATCC), the FP-2013 strain has been isolated by a co-author of the present study (Dr. D. Musso), and we confirmed the identity of the U-1962 strain by sequencing of the envelope gene.

### Cell culture and primary cell isolation

Vero cells (CCL-81) were obtained from ATCC and maintained in DMEM (Gibco) supplemented with 10% fetal bovine serum (Biochrom). *A. albopictus* C6/36 cells were obained from ATCC and cultured in G-MEM-BHK-21 (Gibco) supplemented with 2% FBS (Biochrom). Human peripheral blood mononuclear cells (PBMCs) were isolated from buffy coats of anonymous healthy blood donors (Interregional blood transfusion SRC Ltd, Bern) by density centrifugation over Ficoll-Paque™ PLUS (GE healthcare). Selection of CD14+ monocytes was done using coated magnetic beads according to the manufacturer’s instructions (Miltenyi Biotec) and seeded at a rate of 1 × 10^6^ cells/ml in RPMI 1640 (Gibco) supplemented with 10% FBS (Gibco), GlutaMax (Gibco), penicillin-streptomycin (Gibco), human GM-CSF (100 ng/ml) and human IL-4 (40 ng/ml) (both from Miltenyi Biotec) for six days at 37 °C, 5% CO_2_. The cultures were fed with fresh cytokines at day three post-isolation.

### ZIKV infections

For infection, Vero, C6/36 cells and MoDCs were seeded in tissue culture plates 24 h prior to infection with ZIKV. Cells were washed once with pre-warmed phosphate-buffered saline solution (PBS) (Gibco) and ZIKV, diluted with culture medium without supplements, was added to reach the desired MOI. Based on preliminary experiments with two ZIKV strains in Vero and C6/36 cells, we selected a MOI of 0.1 TCID_50_/cell to perform the African and Asian lineage strain comparison. Indeed, already at 24 h post infection at MOIs < 1 TCID_50_/cell, we detected significant frequency of ZIKV-positive cells and high titers of live virus in supernatants. The virus was adsorbed for one hour at 37 °C or 28 °C. Next, the inoculum was discarded and the cells were washed three times with pre-warmed PBS. Fresh culture medium was added to each well and the cells were incubated at 37 °C or 28 °C, 5% CO_2_ until harvesting. As controls, the culture supernatant from uninfected C6/36 cells was used for mock infection and Poly(IC) (30 µg/ml) (Sigma-Aldrich) in combination with Resiquimod (10 µM) (Sigma-Aldrich), Poly(AU) (0.1 µg/ml) (Sigma-Aldrich) and 5′ppp-dsRNA (1 µg/ml) (InvivoGen) were used as TLR ligands.

### RNA harvesting and quantitative RT-PCR

Total RNA was extracted from cell lysate using NuceloSpin® RNA II filtered columns according to the manufacturer’s protocol (Macherey-Nagel). Complementary DNA (cDNA) was generated using the Omniscript-RT Kit (Qiagen) using random hexamers primers (Invitrogen). For quantitation of RNA expression levels, quantitative RT-PCR was performed using TaqMan® Fast Universal PCR Master Mix (Applied Biosystems) according to the manufacturer’s instructions. The primer and probe sequences used to detect ZIKV, IFNs, ISGs, and the housekeeping control 18S RNAs have been previously described^50–52^. RNA expression levels of ZIKV, IFN type I and III, and the 18 S housekeeping gene were determined by absolute quantification by the serial dilution of plasmids containing the cDNA of the gene of interest. The results were expressed as RNA copies per 10^12^ 18S copies. The analysis of the RNA expression levels of the ISGs normalized to 18S was done by using the ∆∆Ct method^53^. The strategy used for quantification of sfRNA level was previously described by Manokaran *et al*.^30^ Briefly, we used one pair of primers and one probe recognizing specifically sfRNA (sfRNA-FW: 5′-CCCAGGAGAAGCTGGGAAAC-3′, sfRNA-RV: 5′-GCCACCTTC TTTTCCCATCCTG-3′, sfRNA probe 5′-FAM-GAAGAAGCCATGCTGCCTGT-BHQ1-3′) and a second pair of primers and another probe for gRNA (gRNA-FW: 5′-CGYTGCCCAACACAAGG-3′, gRNA-RV: 5′-CCACYAAYGTTCTTTTGCABACAT-3′, gRNA probe 5′-FAM-AGCCTACCTTGAYAAGCARTCAGACACYCAA-BHQ1-3′). We normalized the sfRNA and gRNA ratios to the 18S housekeeping gene. For the amplification on ABI Fast 7500 Sequence Detection System (Applied Biosystems), the following conditions were used: 95 °C 20 sec, 40 cycles of the following: 95 °C 3 sec, 60 °C 30 sec, real-time data were analyzed using the SDS software (Applied Biosystems).

### Titration of viruses with tissue culture infectious dose 50% assay

Cell supernatants were collected 24, and 48 h p.i. and stored at −70 °C. Different 10-fold dilutions of cell supernatants were spread onto Vero cells and maintained for 72 h at 37 °C, 5% CO_2_. Next, the cells were washed with PBS, fixed with 4% paraformaldehyde (PFA) and incubated with anti-flavivirus group antibody 4G2 (ATCC, HB-112™) in PBS supplemented with 0.3% saponin (Sigma-Aldrich), followed by horseradish peroxidase-conjugated goat anti-mouse antibody (Dako) incubation for 30 min and staining with 3-amino-9-ethylcarbazole substrate (Sigma-Aldrich). Titers expressed as TCID_50_/ml were determined by using the Reed and Muench method^54^.

### Lactate dehydrogenase assay

Cell supernatants were collected 24, and 48 h p.i. and stored for short term at −20 °C. The lactate dehydrogenase activity of each sample was measured in triplicate according to a previously released protocol^39^.

### Flow cytometry

The following conjugated mouse monoclonal antibodies were used: APC-CD197 (HLA-DR, Biolegend), APC-CD119 (CCR7, eBioscience), CD40 (Bp50, ATCC), CD80/86 (Ancell). The following antibodies have been used for the phenotyping of MoDC cultures: PerCP-CD3 (clone SK6), PerCP-Cy™5.5-CD56 (clone B159), PerCP-CD19 (clone 4G7), FITC-CD14 (clone MφP9), and PE-CD11c (clone 3.9; all from BioLegend). ZIKV-infected cells were detected using the anti-flavivirus group antibody 4G2. ZIKV-induced cell death was measured with the LIVE/DEAD^®^ Fixable Aqua Dead Cell stain kit (Thermo Fisher) according to the manufacturer’s instructions. Data was acquired on a FACS Canto flow cytometer (BD Bioscience) and analyzed with the FlowJo software (Tree Star).

### Confocal imaging

DCs were infected with ZIKV and plated on Lab-Tek^®^II coated with fibronectin (BD Bioscience). At 24 and 48 h p.i., ZIKV-infected and mock-treated cells were fixed with 4% PFA. Cells were permeabilized with 0.3% saponin and stained intracellularly for ZIKV with the anti-flavivirus group 4G2 antibody. Cells were incubated with Ax-488-conjugated anti-mouse IgG2a antibody (Molecular Probes). The cells where washed once with PBS prior incubation for 5 min at 37 °C with DAPI dye (Sigma). The slides were mounted with Mowiol 4–88 (Sigma). For confocal microscopy analysis, a Nikon confocal microscope A1 (Nikon) combined with an ECLIPSE Ti inverted microscope (Nikon) and a digital imaging Nikon software (NIS-Elements AR 3.30.02) were used. The image acquisitions were performed at 20X magnification; in order to give high-resolution images, the acquiring setting was performed with optimized voxel size and automatic threshold. The images were analyzed with Imaris 8.0.2 software (Bitplane AG). To avoid false-positive emissions, different settings were applied including background subtraction, threshold applications, gamma correction, and maxima.

### Phylogenetic analysis

Tree Builder module of Geneious software (Biomatters Ltd, Auckland, New Zealand) was used to produce a phylogenetic tree using the MrBayes plugin (Bayesian phylogenetic inference using Markov chain Monte Carlo-based method^55^). For this purpose, Spondweni virus strain AR 94 was used as the outgroup to root the tree.

### Statistical analysis

The GraphPad Prism 6 software (GraphPad software, La Jolla, CA USA) was used for statistical analysis. Multiple comparison was performed with a one-way ANOVA and the Tukey post-hoc test. A p value < 0.05 was considered statistically significant.

## Electronic supplementary material

Supplementary information
