# Supplementary material for: Silent infection of human dendritic cells by African and Asian strains of Zika virus
Source: Sci Rep. 2018 Apr 3;8:5440. doi: 10.1038/s41598-018-23734-3 (PMC5882923; doi:10.1038/s41598-018-23734-3)
Supplement: Supplementary file 1 — Supplementary information [file 41598_2018_23734_MOESM1_ESM.pdf]

Silent infection of human dendritic cells by African and Asian strains of Zika virus

Nathalie J. Vielle[1,2], Beatrice Zumkehr[1], Obdulio García-Nicolás[1], Fabian Blank[3], Miloš Stojanov[4], Didier Musso[5], David Baud[4], Artur Summerfield[1,6], and Marco P. Alves[1,6, #]

Supplementary information

Supplementary Figure 1

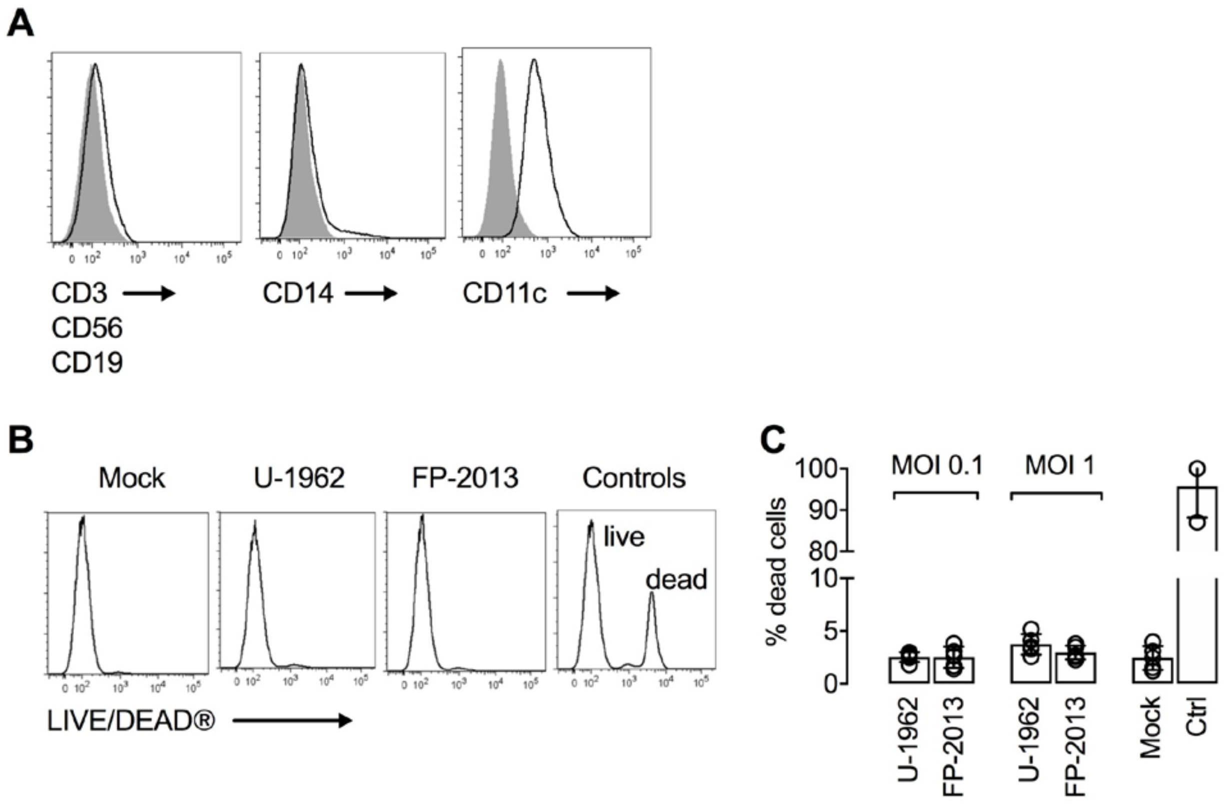

Fig. S1. **Phenotyping of MoDCs and cell death.** (A) Expression levels of cell surface markers measured by flow cytometry in MoDCs Mock-treated or infected with FP-2013 or MP1751 at an MOI of 0.1 and 1 $TCID_{50}$/cell. The data shown are representative of MoDCs generated from 3 independent blood donors. (B) ZIKV-induced cell death of MoDCs Mock-treated or infected with FP-2013 or MP1751 at an MOI of 1 $TCID_{50}$/cell assessed with a LIVE/DEAD® assay by flow cytometry. The data shown are representative of MoDCs generated from 5 independent blood donors. (C) Percentages of dead cells measured by flow cytometry with a LIVE/DEAD® assay 24h after Mock-treatment or infection with FP-2013 or MP1751 at an MOI of 0.1 and 1 $TCID_{50}$/cell. Data are presented as mean of DCs generated from 5 independent blood donors.

Supplementary Figure 2

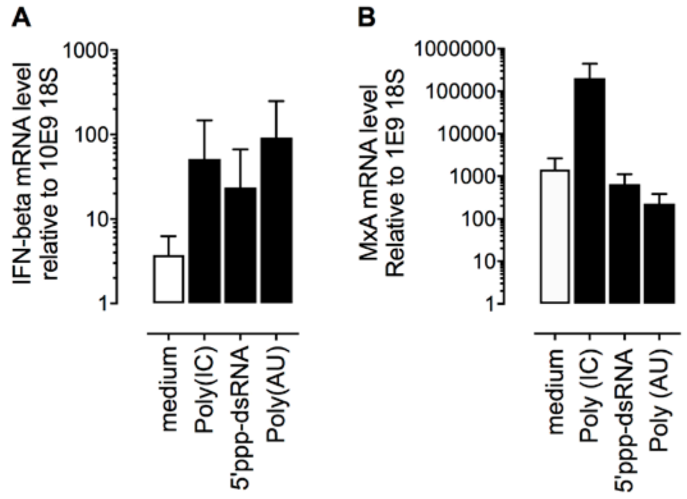

Fig. S2. Screening of TLRs and RLRs ligands for IFN pathway induction in MoDCs. Measurement of IFN-β (A) and MxA (B) expression level relative to 18S by quantitative RT-PCR in MoDC cultures 48h p.i. after stimulation with Poly(IC) 10ug/ml, 5'ppp-dsRNA 1 ug/ml and Poly(AU) 0.1 ug/ml. Data are presented as mean +/- SD of DCs generated from 4 independent blood donors.

Supplementary Figure 3

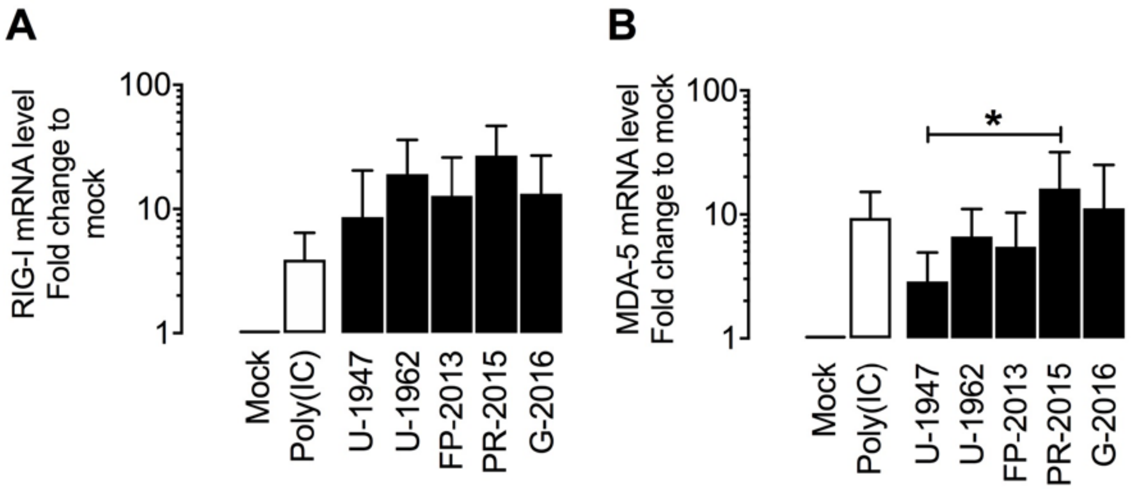

Fig. S3. mRNA levels of RLRs upon poly(IC) stimulation and ZIKV strains infection. Fold change of gene expression levels relative to mock of RIG-I (A) and MDA-5 (B) measured by quantitative RT-PCR in MoDCs 48h p.i. with five ZIKV strains at a MOI of 0.1 TCID50/cell and after addition of Poly(IC). Data are presented as mean +/- SD of MoDCs generated from 4-15 independent blood donors, * $p<0.05$.

Supplementary Figure 4

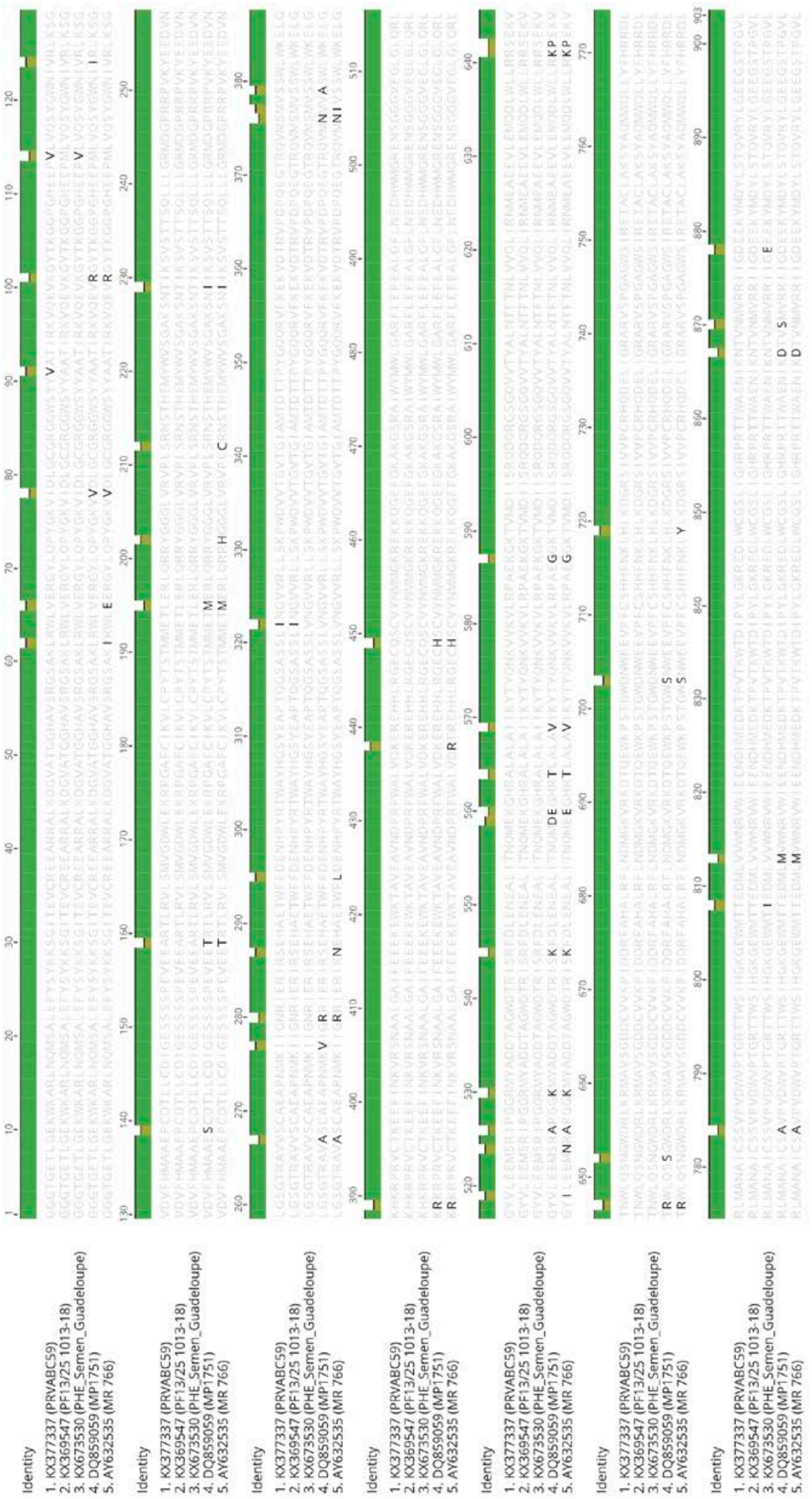

Fig. S4. Alignment of full-length NS5 proteins of the strains included in the study. Where present, disagreements between strains are highlighted in black, while residues in common are depicted in light grey. Sequences were aligned using the ClustalW2 module of Geneious software (Biomatters Ltd.) with default parameters.
